# Supplementary material for: Predicting instabilities in transient landforms and interconnected ecosystems
Source: Nat Commun. 2026 Feb 6;17:1316. doi: 10.1038/s41467-026-68944-w (PMC12881616; doi:10.1038/s41467-026-68944-w)
Supplement: Supplementary file 1 — Supplementary Information [file 41467_2026_68944_MOESM1_ESM.pdf]

Supplement to: Predicting Instabilities in Transient Landforms and Interconnected Ecosystems

Taylor Smith[1], Andreas Morr[2,3], Bodo Bookhagen[1], Niklas Boers[4,3,5]
[1]Institute of Geosciences, Universität Potsdam, Germany
[2] Department of Mathematics, School of Computation, Information and Technology, Technical University of Munich, Germany
[3]Potsdam Institute for Climate Impact Research, Germany
[4] Munich Climate Center and Earth System Modelling Group, Department of Aerospace and Geodesy, TUM School of Engineering and Design, Technical University of Munich, Germany
[5]Department of Mathematics and Global Systems Institute, University of Exeter, UK

Corresponding author:
Taylor Smith
Email: tasmith@uni-potsdam.de

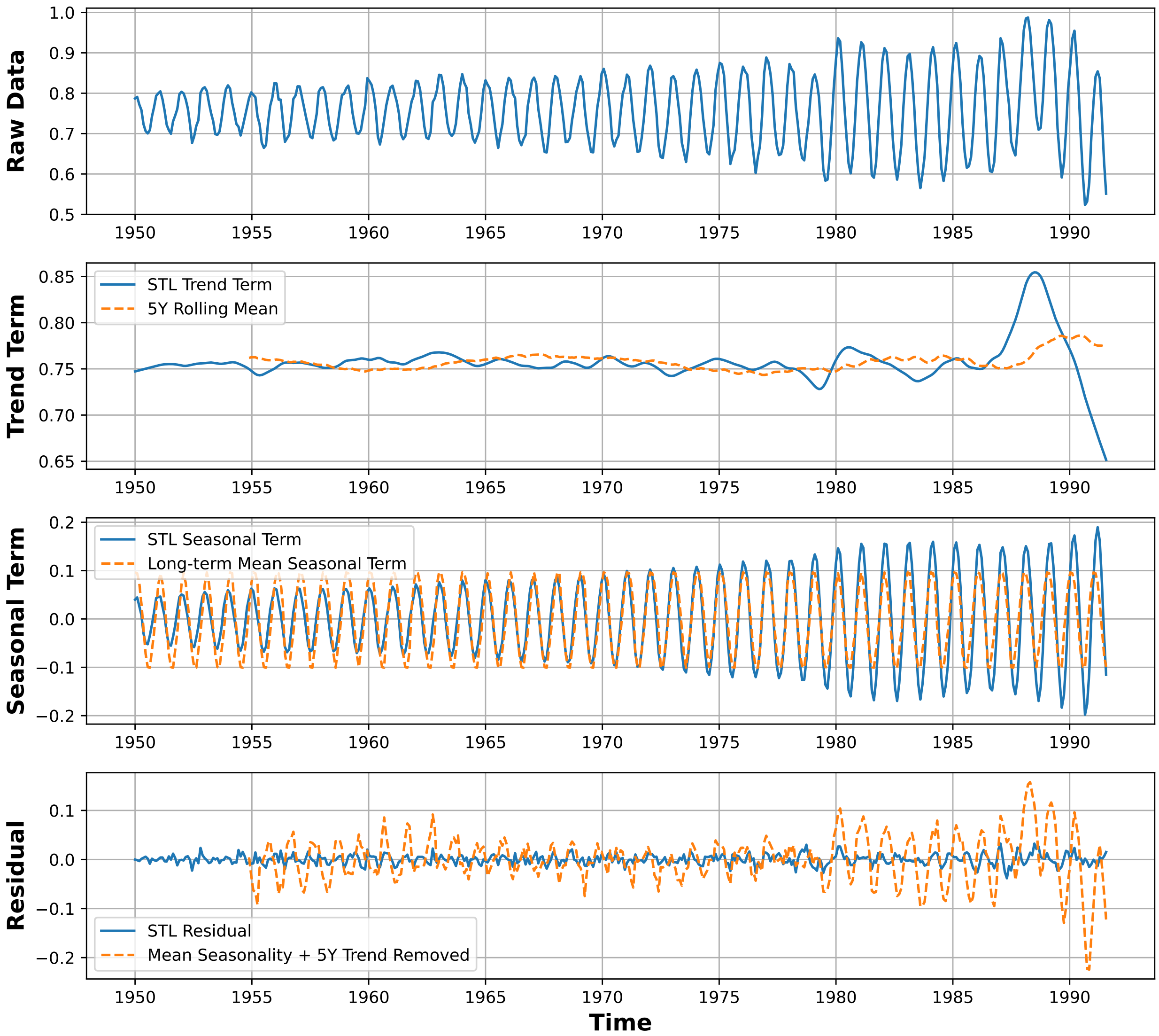

Supplementary Figure S1: STL Deseasoning (blue) and simple mean-based deseasoning/detrending (orange) on synthetic data from Supplemental Figure S14 (up to the transition point). (A) Raw data, (B) trend term, (C) seasonal term, (D) residual term. There is significant seasonality left in the STL trend term, and far more variability in the STL seasonal amplitude than in the real (fixed) seasonal amplitude of the data. Both methods are designed to produce a residual without seasonal or long-term trend influences, with different underlying assumptions about the stability of the trend and seasonality.

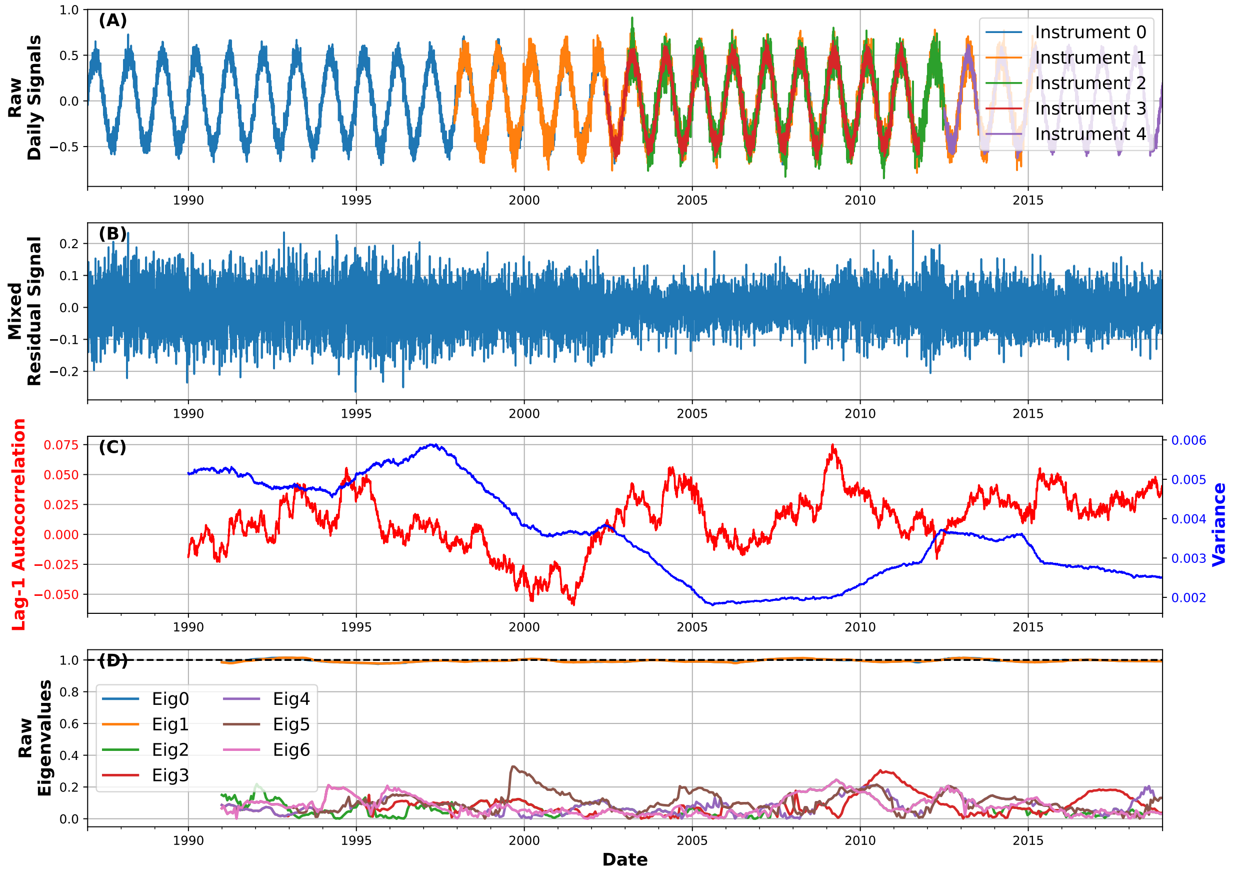

Supplementary Figure S2: (A) Synthetic signals with varying noise levels and overlapping time periods. (B) Mixed daily average, deseasoned and detrended with STL. (C) AC1 and variance computed on the deseasoned residual, showing spurious changes due to noise level changes. (D) Eigenvalues computed on the daily average signal without deseasoning or detrending, showing that the stable cycle is captured (strong eigenvalue near 1) and the noise changes are partitioned into low-energy eigenvalues. There are no spurious high-energy eigenvalues induced by noise-level changes.

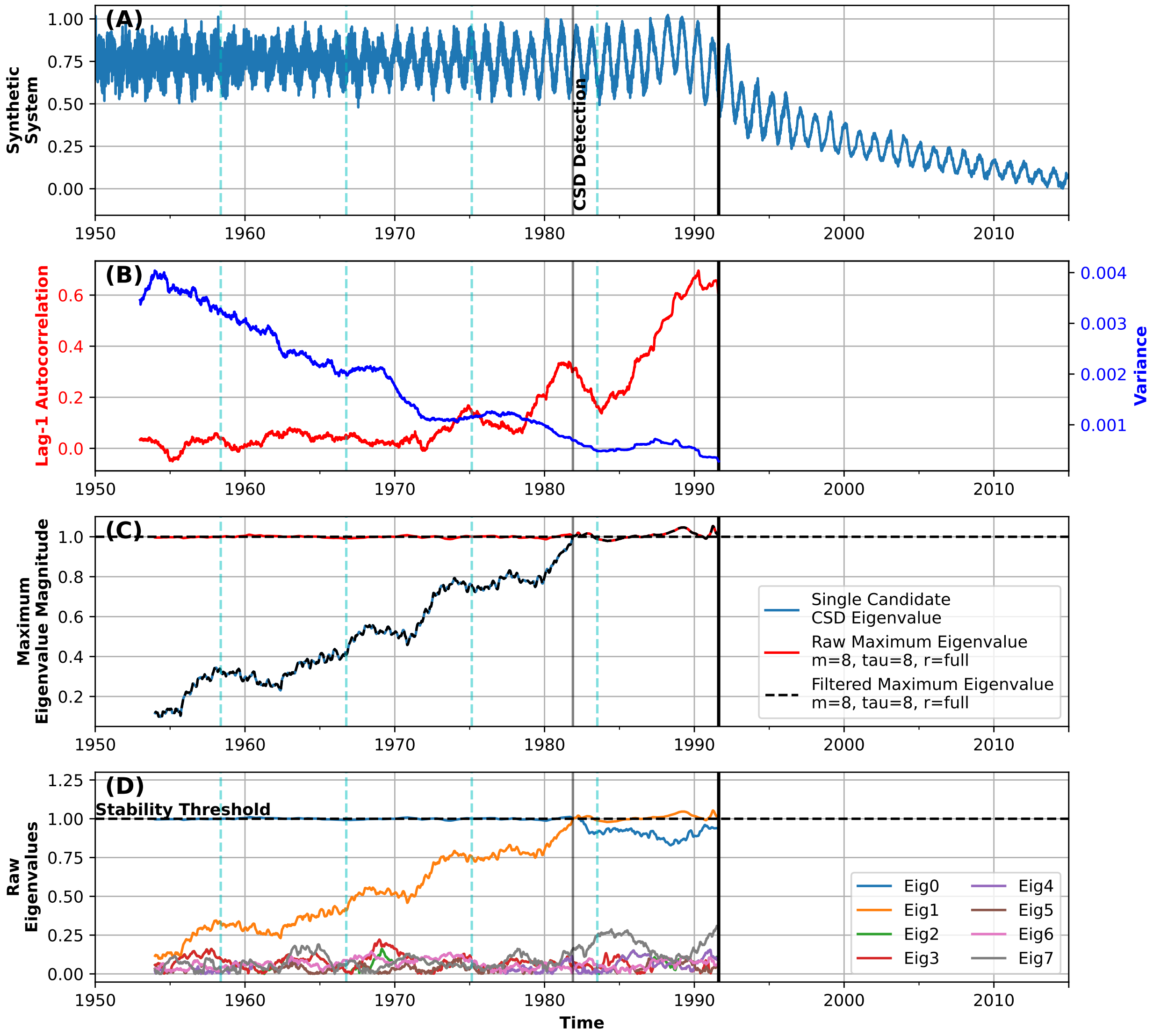

Supplementary Figure S3: (A) Synthetic system with decreasing additive noise levels, marked at cyan dashed vertical lines. Vertical gray line marks instability detection from eigenvalue tracking. (B) Changes in AC1 and variance due to noise level shifts in deseasoned and detrended residual. (C) Maximum and filtered maximum eigenvalue, showing expected CSD signal without bias. (D) All eigenvalues computed on the raw, noise-varying system.

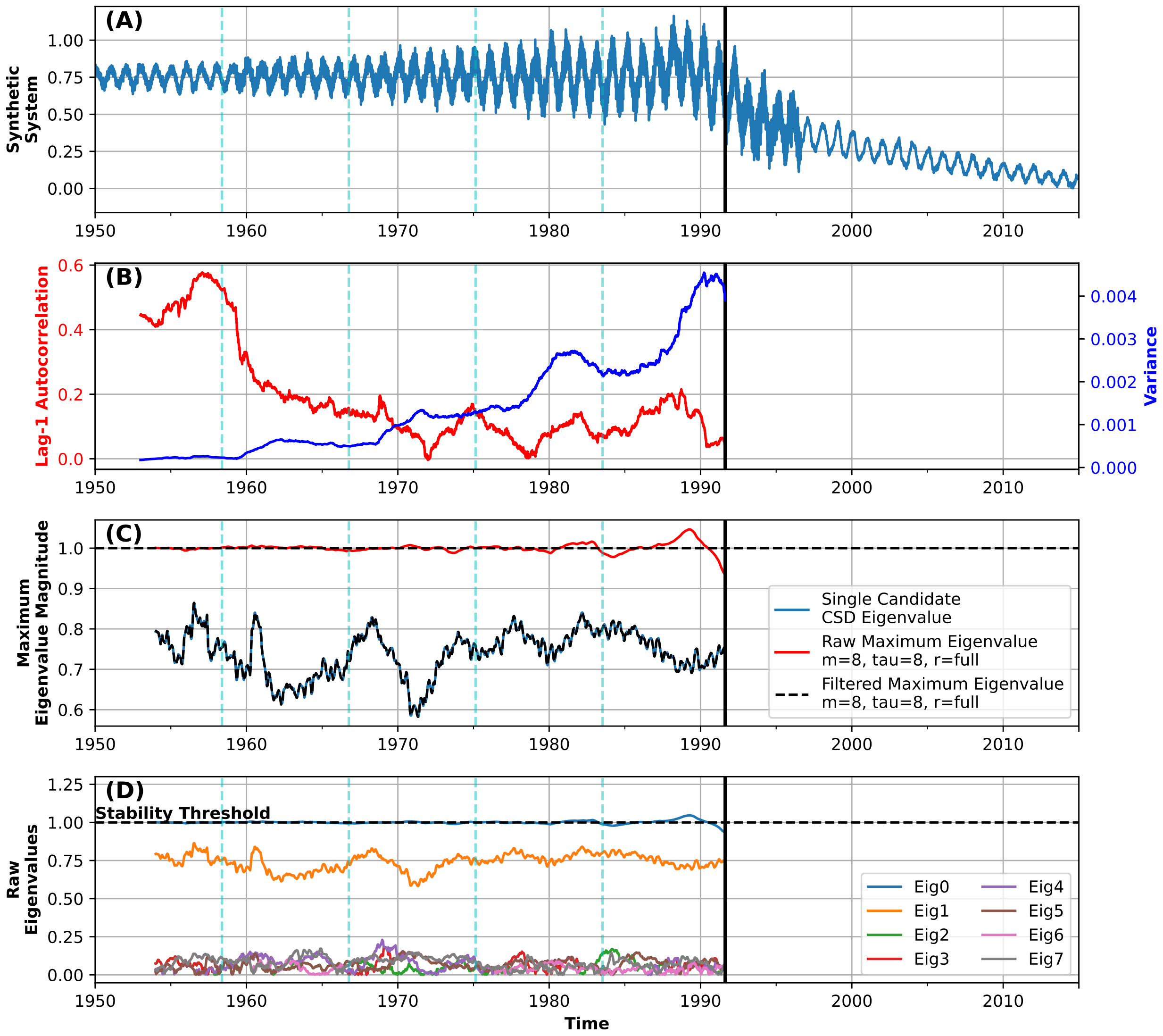

Supplementary Figure S4: (A) Synthetic system with increasing additive noise levels, marked at cyan dashed vertical lines. (B) Changes in AC1 and variance due to noise level shifts in deseasoned and detrended residual. (C) Maximum and filtered maximum eigenvalue, showing a missed CSD signal due to high noise around the transition. (D) All eigenvalues computed on the raw, noise-varying system.

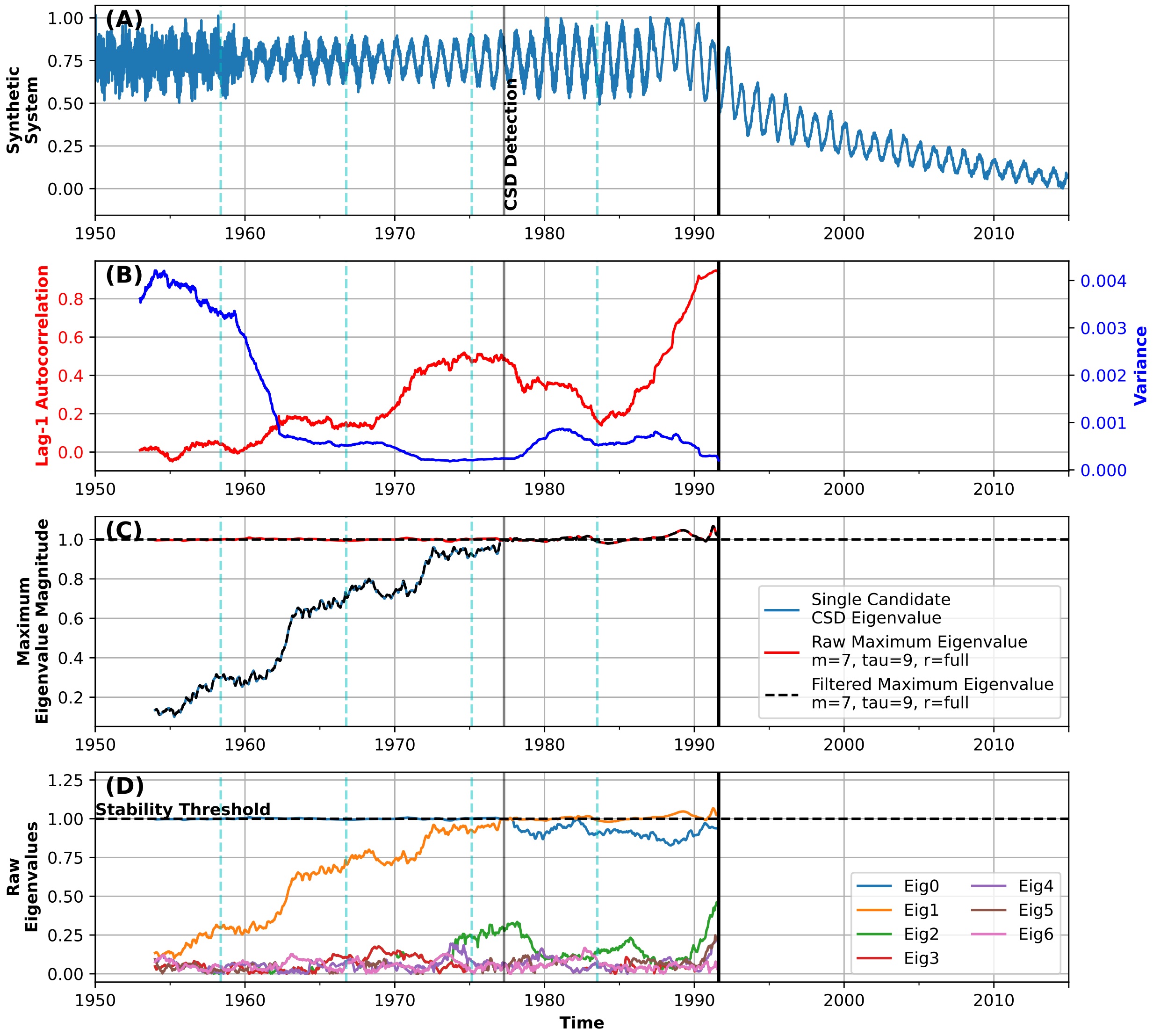

Supplementary Figure S5: (A) Synthetic system with shuffled additive noise levels, marked at cyan dashed vertical lines. Vertical gray line marks instability detection from eigenvalue tracking. (B) Changes in AC1 and variance due to noise level shifts in deseasoned and detrended residual. (C) Maximum and filtered maximum eigenvalue, showing expected CSD signal without bias. (D) All eigenvalues computed on the raw, noise-varying system.

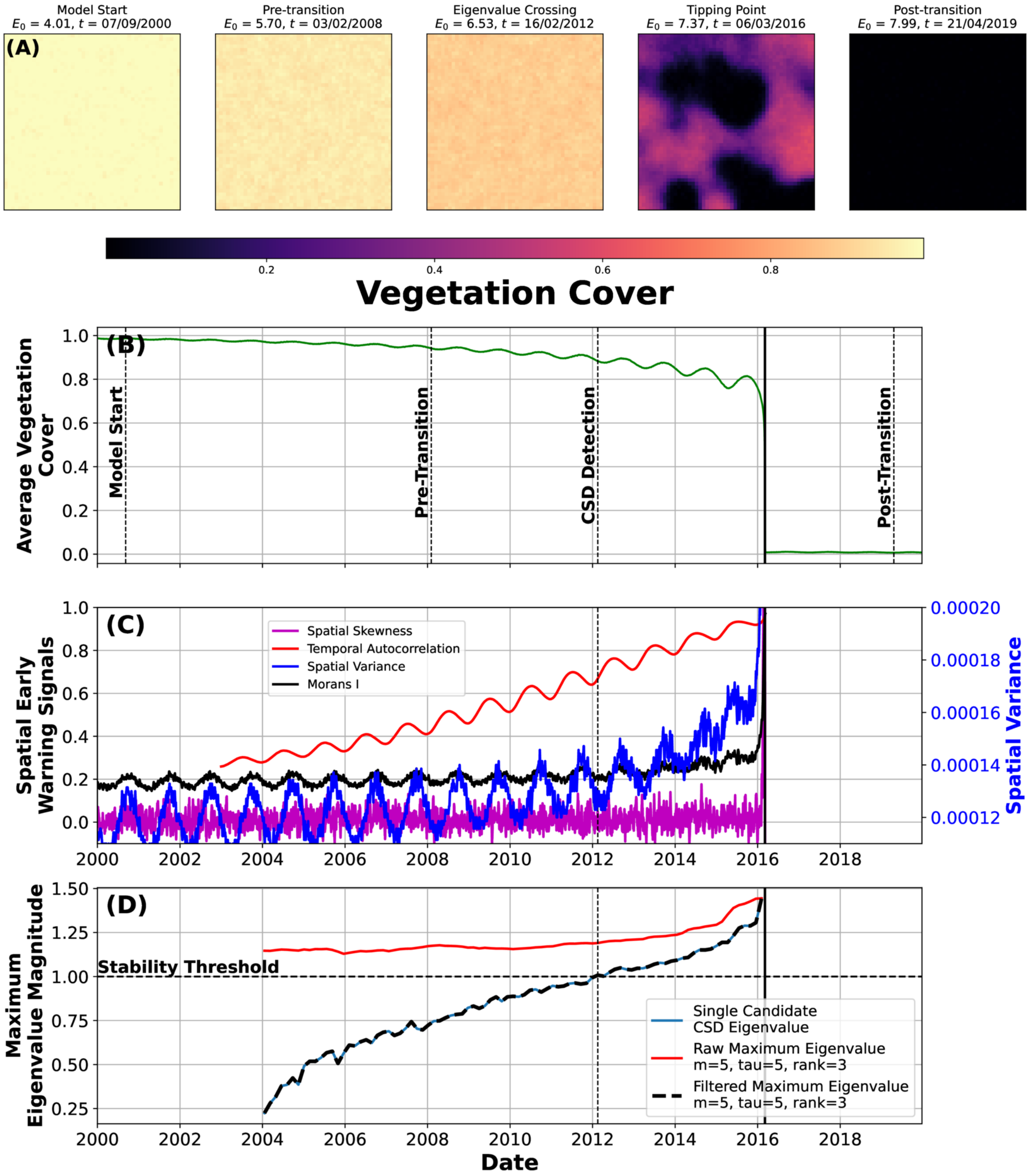

Supplementary Figure S6: Spatial vegetation tipping model. (A) Slices from the spatial grid of the vegetation model. E_0 quantifies environmental stress (Methods). (B) Mean vegetation state, with vertical lines showing the location of spatial snapshots from (A). (C) Typical spatial early-warning signals on raw seasonal data: spatial skewness (purple), temporal autocorrelation (red), spatial variance (blue), and Moran's I (black). The application of these indicators on the raw time series data is not substantiated by critical slowing down theory; changes in these statistics are strongly biased by seasonal fluctuations and changes in the system mean state. (D) Eigenvalue magnitudes, showing a crossing of the dominant non-seasonal eigenvalue well before the transition. The seasonal eigenvalue is slightly unstable (magnitude >1) due to the slow increase in seasonal amplitude in the model as the resilience of the system decreases, which drives the seasonal amplitude slightly higher through time.

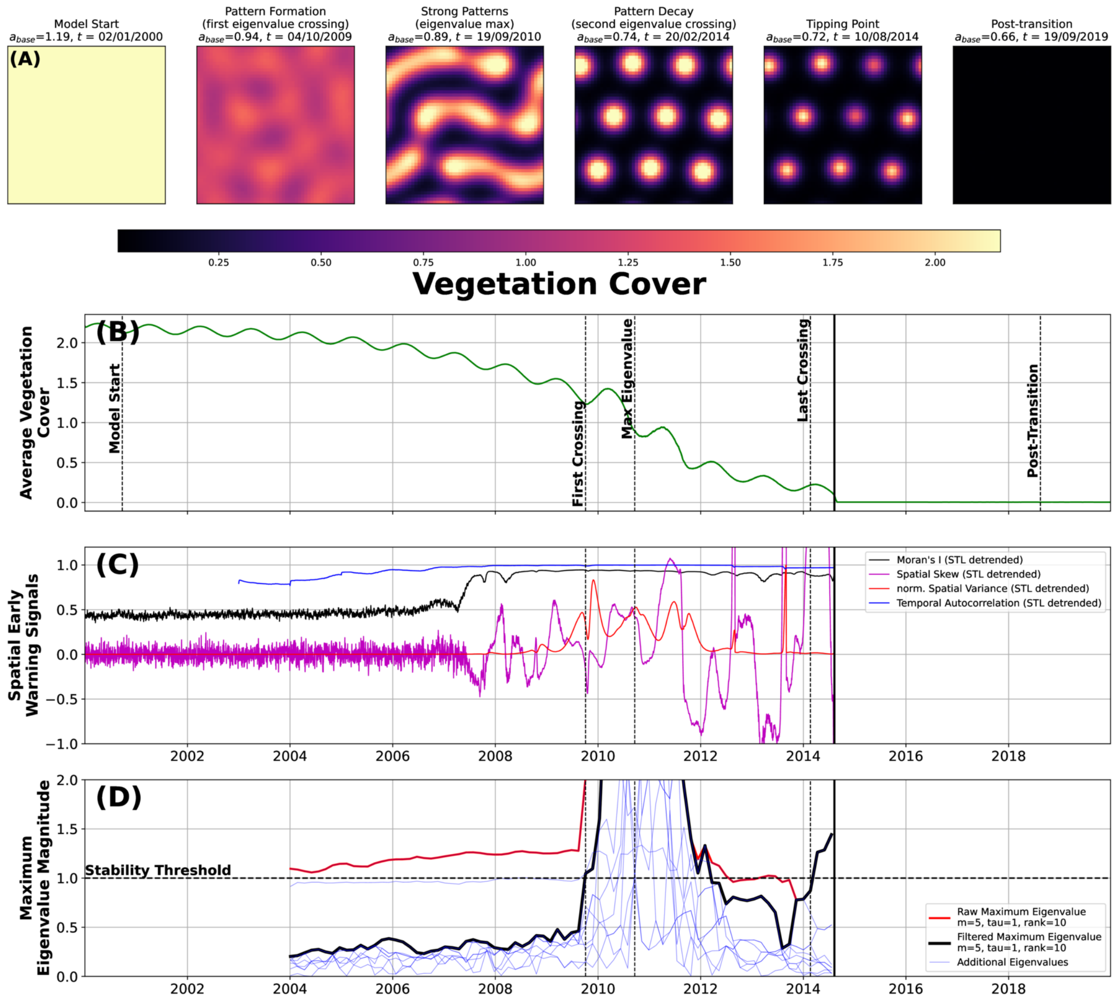

Supplementary Figure S7: Eigenvalue tracking on a simple pattern-formation model with seasonality. (A) Spatial maps of snapshots in time linked to different points in the time series. a_{base} quantifies environmental conditions (Methods). (B) Mean vegetation state, with vertical lines showing the location of spatial snapshots from (A). (C) Spatial early warning signals (on data detrended/deseasoned with STL) have different responses to the onset of patterns and the collapse of the system. (D) Eigenvalue tracking, showing a strong and slightly unstable seasonal eigenvalue as well as an increase in the dominant non-seasonal eigenvalue up until the first one-crossing point. There are also high and sustained eigenvalue magnitudes during the pattern formation period, followed by a decrease as patterns decay. Final short increase in eigenvalue magnitude above one before collapse (marked as last crossing in A,B).

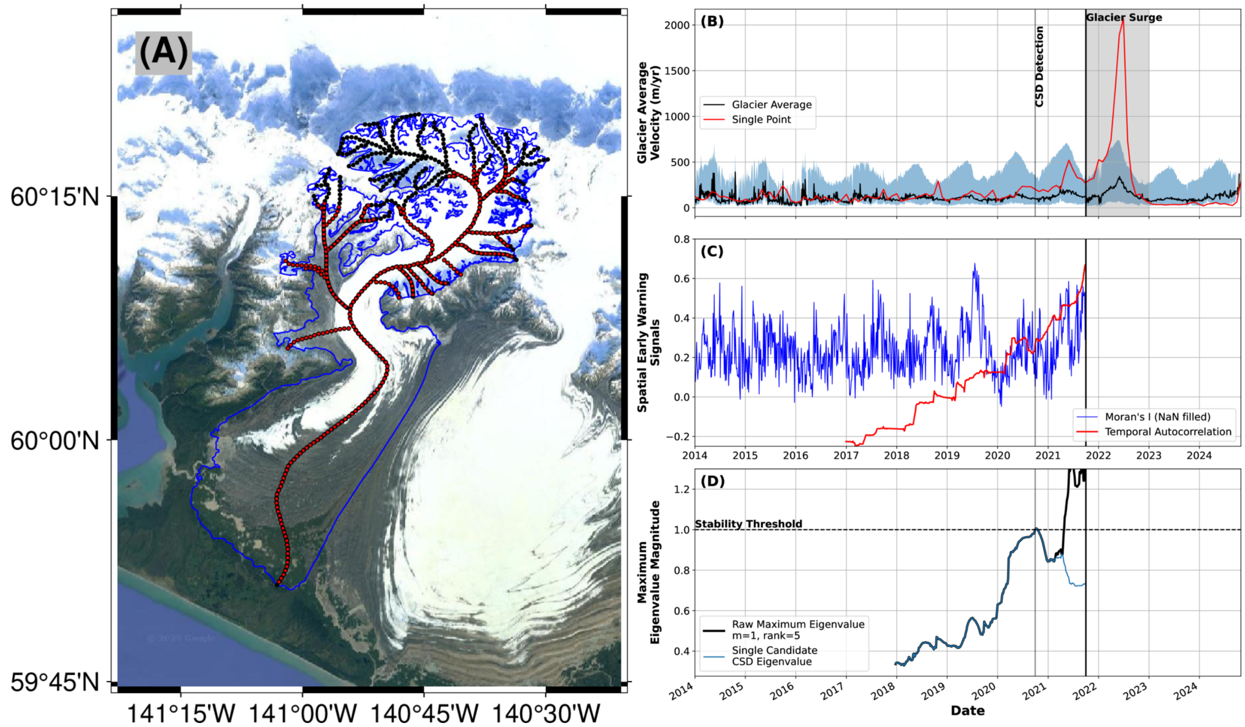

Supplementary Figure S8: (A) Map of target glacier (RGI2000-v7.0-G-01-13271, [-140.847,60.089]) with outline (blue) and sample points ($n = 337$) along the glacier centerline (red, low-elevation points, black, all centerline points). Background from Google Earth. (B) 4-day ($t = 1004$ time steps) glacier-average velocity (black) plus 25th-75th percentile range (shaded blue), alongside single strongly surging point (red). Shaded area shows surge period based on velocity magnitude. Vertical gray line marks instability detection from eigenvalue tracking. (C) Spatial early warning signals on deseasoned/detrended velocity data (Methods). (D) Maximum eigenvalue using the set of velocities over all low-elevation centerline points, showing a trend towards instability far before the actual surge despite lack of lag embedding (m=1, r=5).

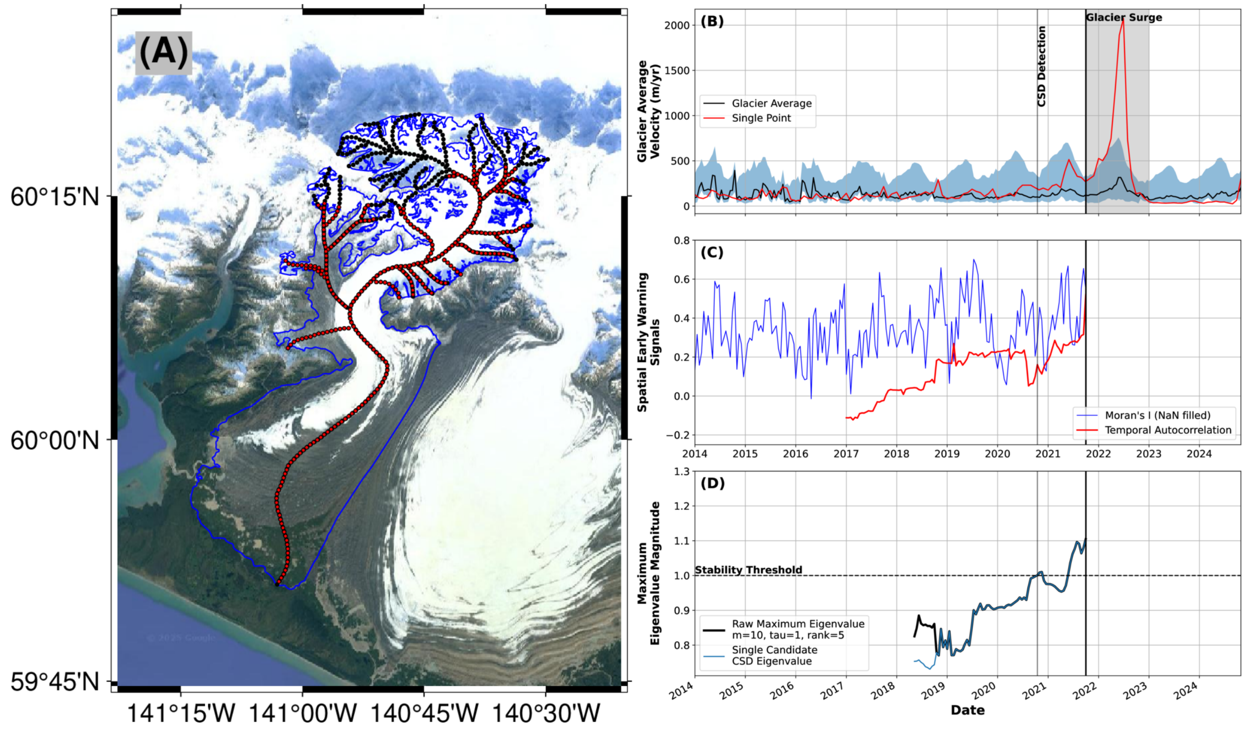

Supplementary Figure S9: (A) Map of target glacier (RGI2000-v7.0-G-01-13271, [-140.847,60.089]) with outline (blue) and sample points ($n = 337$) along the glacier centerline (red, low-elevation points, black, all centerline points). Background from Google Earth. (B) 16-day ($t = 253$ time steps) glacier-average velocity (black) plus 25th-75th percentile range (shaded blue), alongside single strongly surging point (red). Shaded area shows surge period based on velocity magnitude. Vertical gray line marks instability detection from eigenvalue tracking. (C) Spatial early warning signals on deseasoned/detrended velocity data (Methods). (D) Maximum eigenvalue using the set of velocities over all low-elevation centerline points, showing a trend towards instability far before the actual surge.

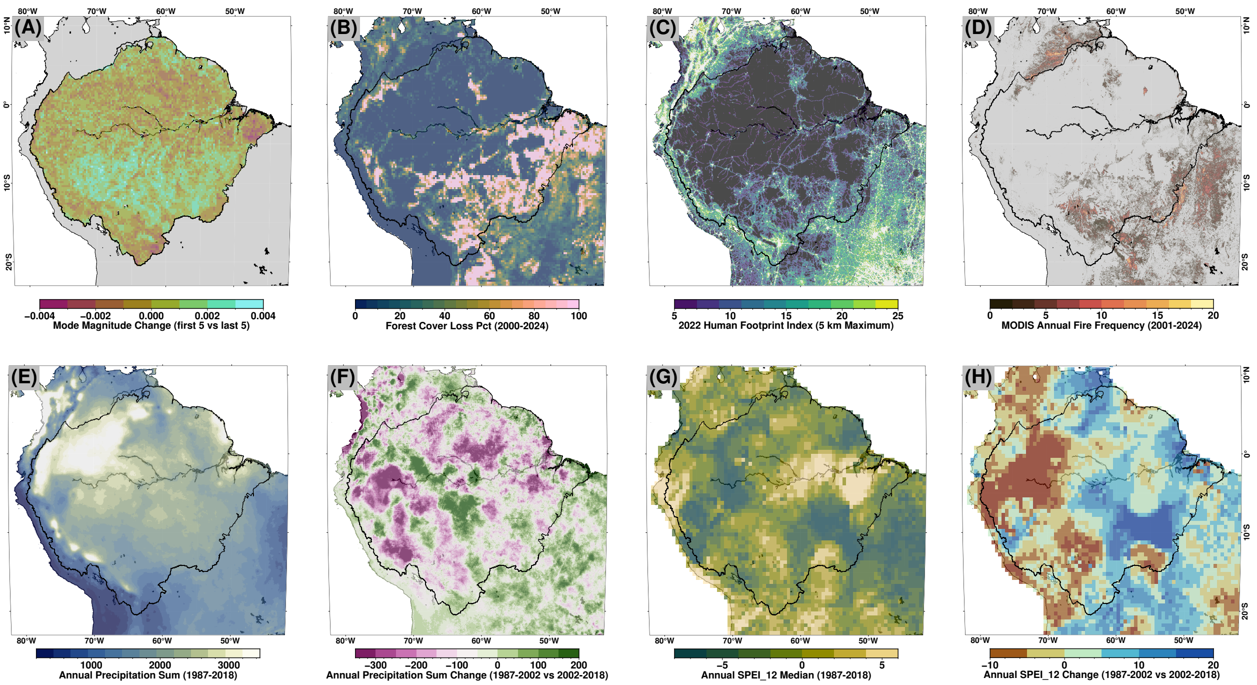

Supplementary Figure S10: Comparison of (A) CSD-Mode Magnitude Difference (c.f. Figure 5) with changes in (B) Forest Cover (2000-2024, summed on a 0.25 dd grid) (Hansen et al., 2015), (C) Human Footprint Index (2022, Mu et al., 2022), (D) MODIS Fire Frequency (2001-2024, Giglio et al., 2021), (E) Annual Precipitation Sum and (F) Sum Change (CHIRPS, Funk et al. 2015), and (G) SPEI Drought Index 12-month Median and (H) Median Change (Vicente-Serrano et al., 2010). While the largest positive mode change (i.e., loss of stability, light blue areas, A) seems to correlate with forest cover loss, it is unlikely that this is the only driving factor.

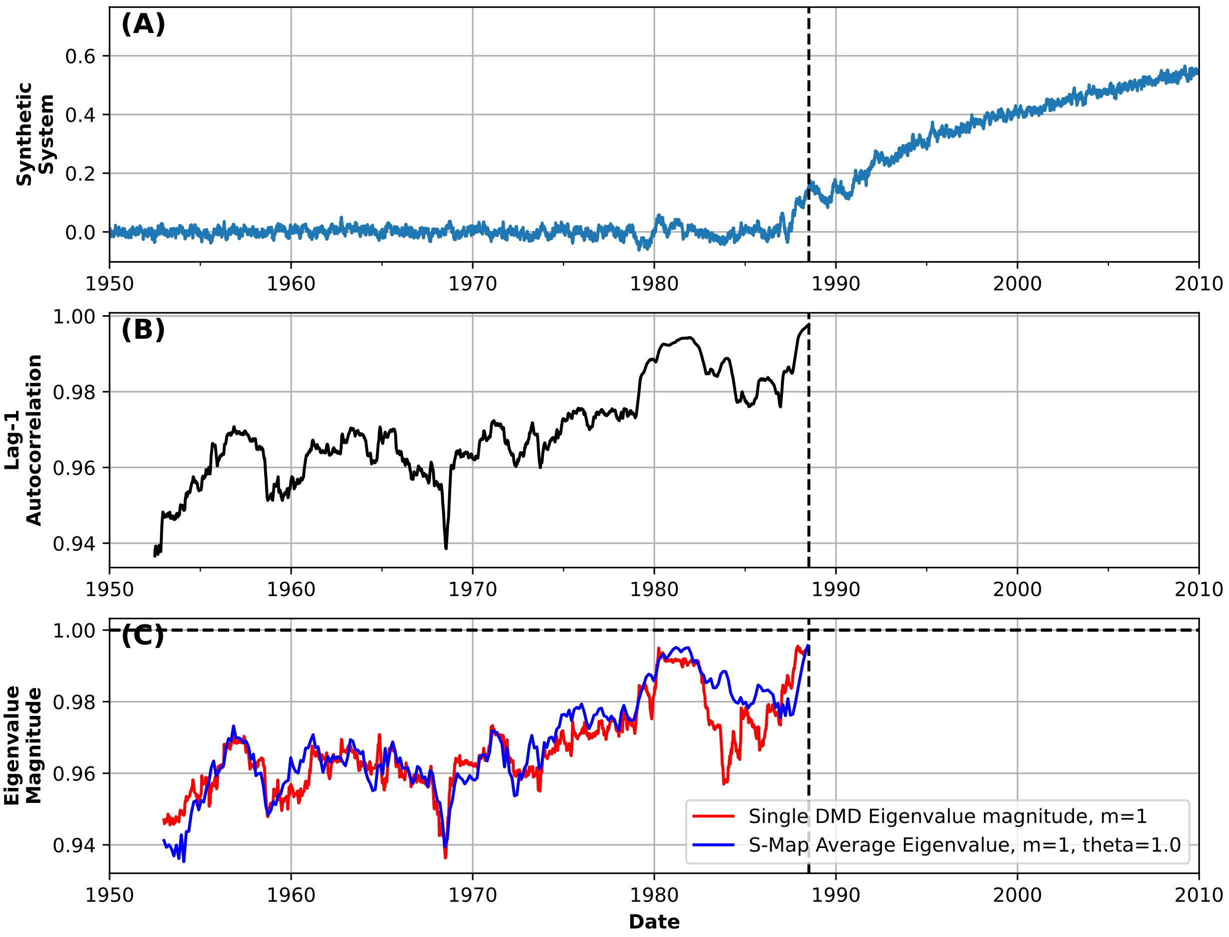

Supplementary Figure S11: (A) Synthetic model showing pitchfork bifurcation without seasonality. (B) Lag-1 autocorrelation over a set of moving windows and (C) DMD and S-Map dominant eigenvalue estimates for the same set of moving windows without lag embedding (Methods).

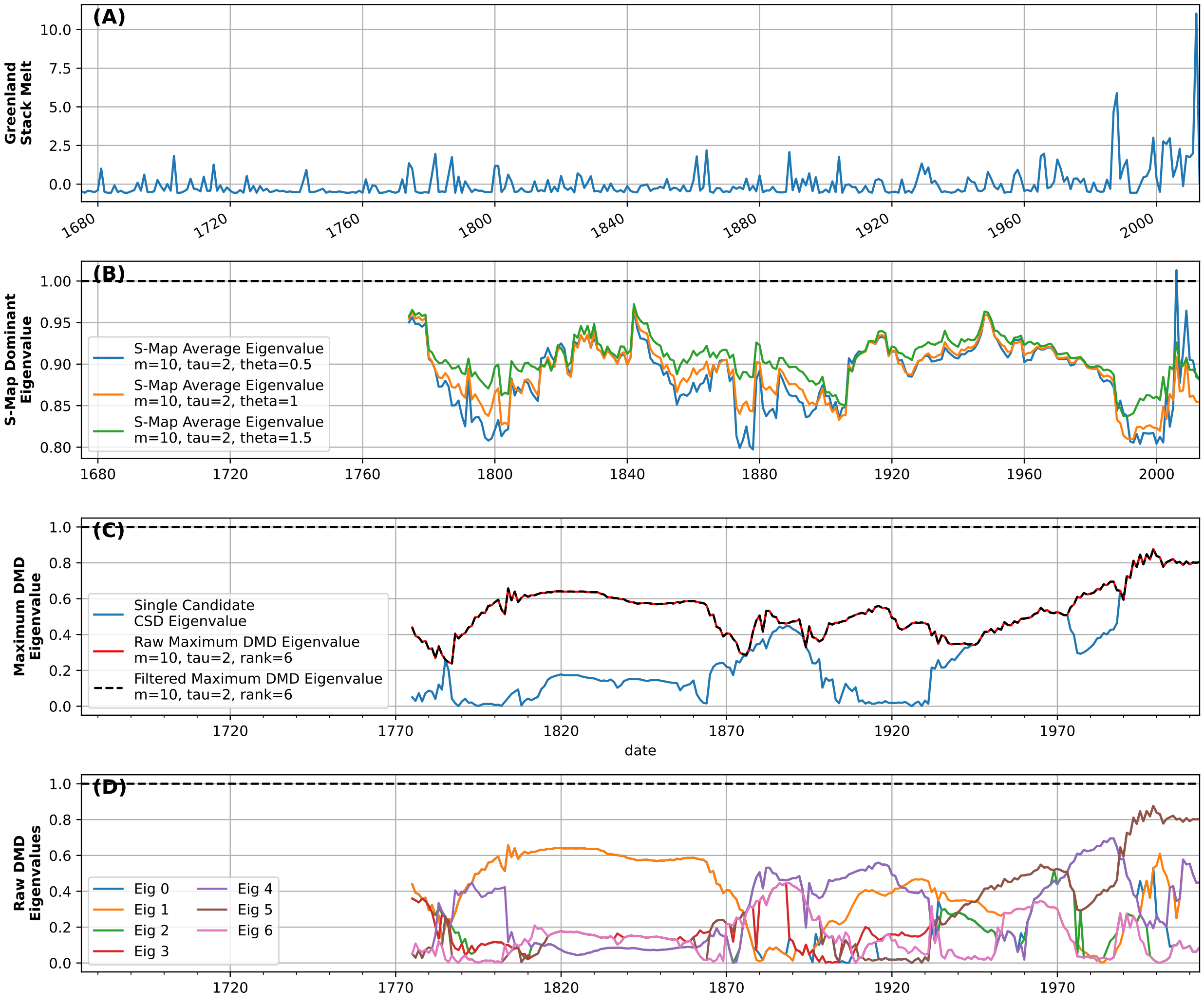

Supplementary Figure S12: (A) Greenland Stack Melt. (B) S-Map eigenvalues for three different theta parameters. (C) Filtered maximum DMD eigenvalue and (D) all DMD eigenvalues.

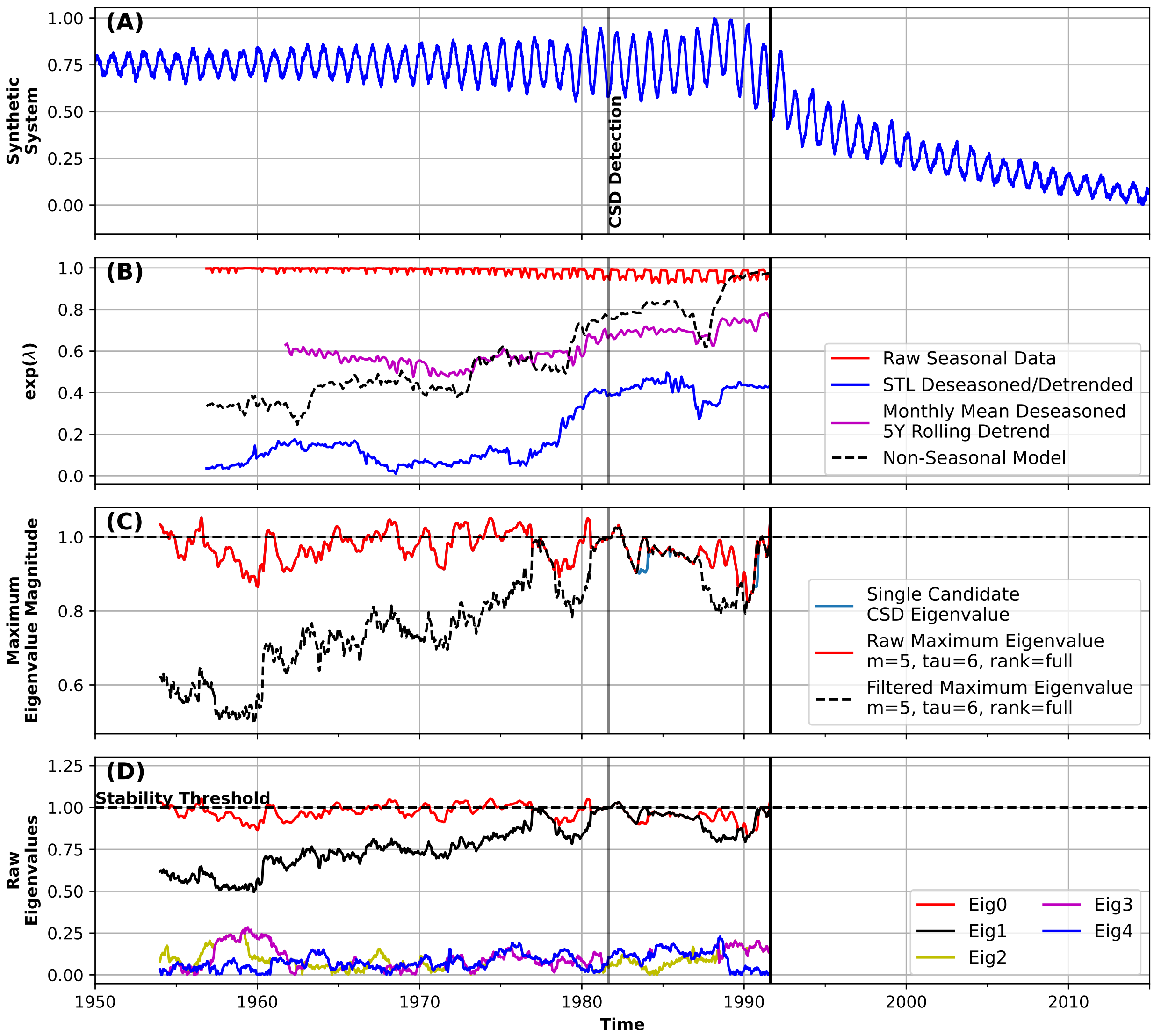

Supplementary Figure S13: (A) Simple pitchfork-bifurcation model with seasonality moving towards a state transition (Methods). Vertical line shows instability detection from eigenvalue tracking. (B) Restoring rate λ (Morr et al., 2024) on non-seasonal and deseasoned/detrended time series. (C) Filtered eigenvalues, showing that a dominant non-seasonal eigenvalue approaches and crosses 1 before the critical transition. The strength of the seasonal eigenvalue has been diminished – but not removed – by mean-centering the data. (D) The set of all eigenvalues shows that most of them capture short-term noise in the system rather than coherent changes.

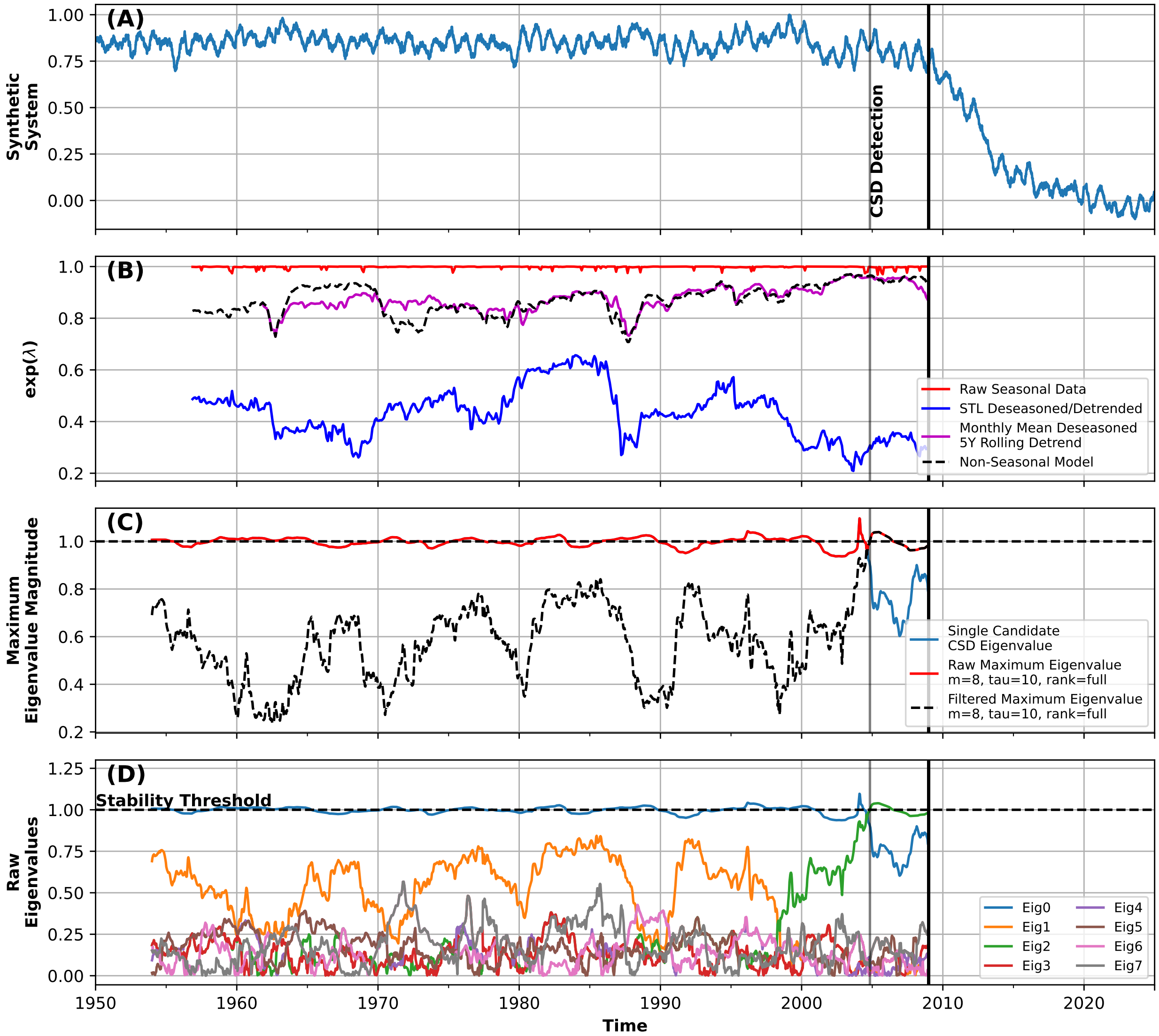

Supplementary Figure S14: (A) Simple pitchfork-bifurcation model with seasonality moving towards a state transition. Forcing is fast and sigmoidal, as opposed to slow and linear in Figure 1. Vertical line shows instability detection from eigenvalue tracking. (B) Restoring rate λ (Morr et al., 2024) on non-seasonal and deseasoned/detrended time series. (C) Filtered eigenvalues, showing that a dominant non-seasonal eigenvalue approaches and crosses 1 before the critical transition. (D) The set of all eigenvalues shows that most of them capture short-term noise in the system rather than coherent changes.

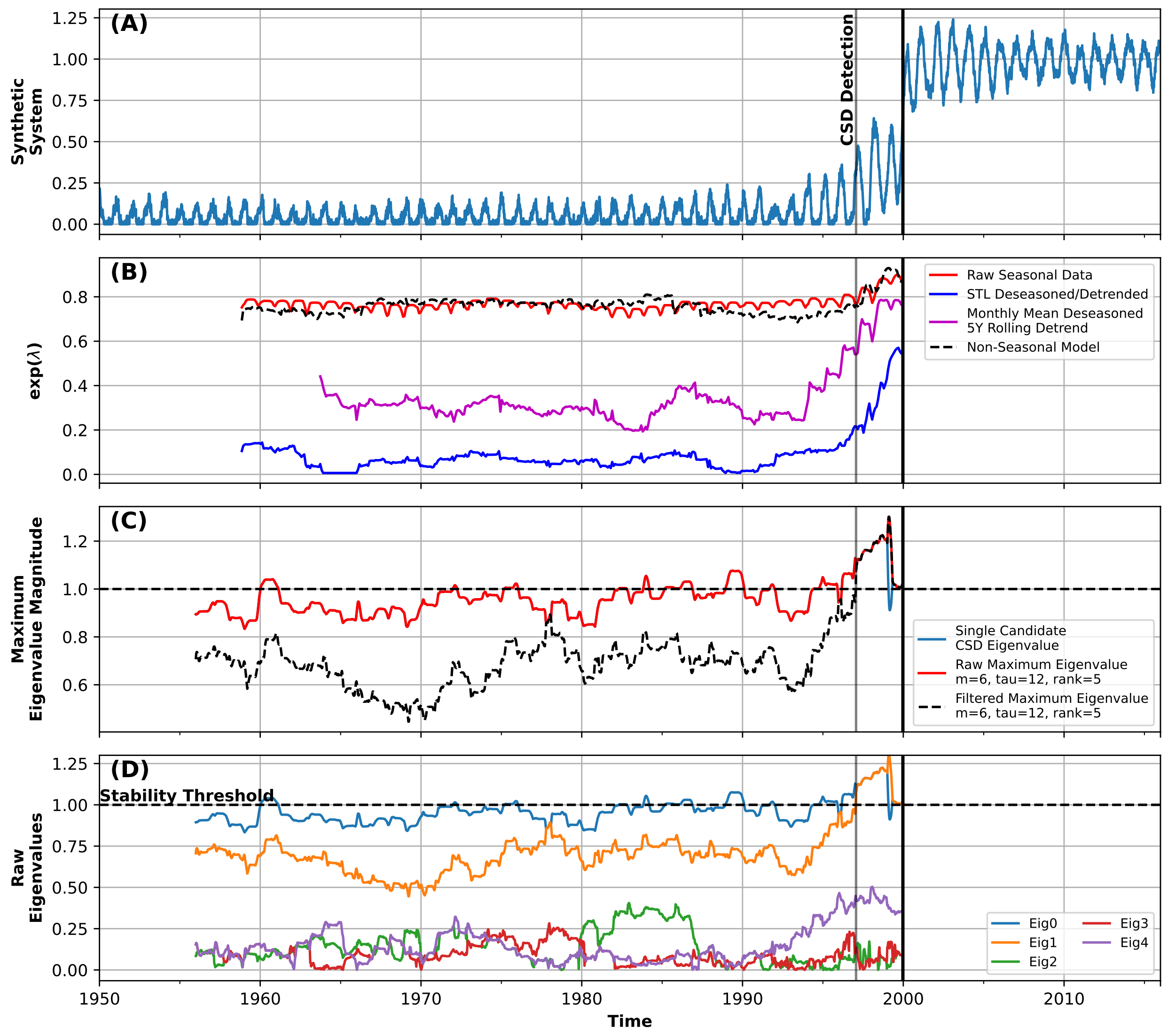

Supplementary Figure S15: (A) Simple logistic model with seasonality moving towards a state transition (Methods). Vertical line shows instability detection from eigenvalue tracking. (B) Restoring rate λ (Morr et al., 2024) on non-seasonal and deseasoned/detrended time series. (C) Filtered eigenvalues, showing that a dominant non-seasonal eigenvalue approaches and crosses 1 before the critical transition. (D) The set of all eigenvalues shows that most of them capture short-term noise in the system rather than coherent changes. Clipping is apparent in λ and eigenvalues due to the imposed lack of negative values in the model.

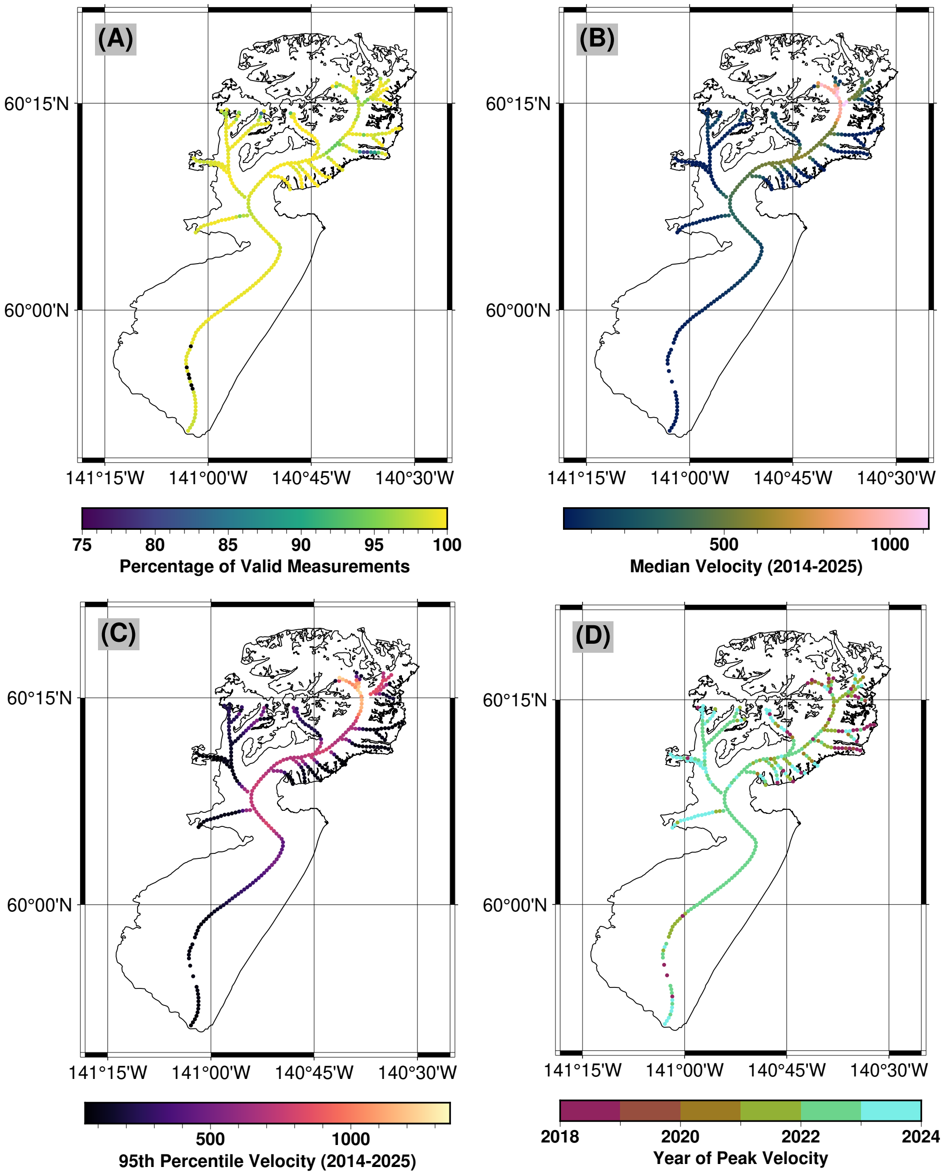

Supplementary Figure S16: Spatial patterns of glacier velocity data for RGI2000-v7.0-G-01-13271. (A) Percentage of available measurements per point. (B) Median velocity over the whole data period. (C) 95th percentile velocity. (D) Year of peak glacier velocity.

Supplemental Tables: Synthetic Model Parameterizations

Time Series Models

For the stationary process model used to test the impact of signal mixing, we roughly mimic the sensor mix used in Vegetation Optical Depth data, following the same methodology as presented in Smith et al. (2023). We use $dt = 0.01$, maintaining daily means as output. This data is used in Supplemental Figure S2.

**Parameter**	**Value / Description**
Time step Δt	0.01 days
Simulation duration	1987–2018
Initial condition x_0	0
Base noise amplitude σ_{base}	0.01
Scaling factor	0.2
Seasonal component	None (residual) or 365 days (seasonal)
Number of signals to mix	5
Signal noise scaling	[1.43, 2.0, 2.29, 1.14, 1.0]
Noise type	Gaussian white noise
Integration scheme	Euler–Maruyama

Table S1: Parameters used in the stationary mixed-signal time series model.

For the pitchfork bifurcation model, we simulate 75 years of data with a $dt = 0.01$, saving the last value per day of the model state. We generate two versions: (1) forced linearly from -0.5 to 0.5 along the whole time axis and (2) with the transition set to occur partway along the simulation period by centering the transition (sigmoidal) on a desired year, and changing the speed of transition by setting the width of the desired transition. We rescale the data to the range [-0.1,1] for display purposes. This data is used in Figure 1 and Supplemental Figures S11,S13 (linear forcing) as well as Supplemental Figure S14 (sigmoidal forcing).

**Parameter**	**Value / Description**
Time step Δt	0.01 days
Simulation duration	1950–2025
Initial condition x_0	0.01
Control parameter range $(p_{\text{start}}, p_{\text{end}})$	$(-0.5, 0.5)$
Target bifurcation year	2005 or None
Transition width	1/5 of total duration or None
Linear damping coefficient	0.0
Noise amplitude σ	0.025
Seasonal amplitude A_{seasonal}	0.05
Seasonal frequency ω	365 day
Integration scheme	Euler–Maruyama
Rescaling range (output)	$[-0.1, 1]$

Table S2: Parameters used in the pitchfork bifurcation time series model.

For the logistic bifurcation model, we simulate 75 years of data with a $dt = 0.1$, saving the last value per day of the model state. We force the transition to occur partway along the simulation time step by centering the logistic function on a desired midpoint. We change the speed of transition by setting the width of the desired transition. This data is used in Supplemental Figure S15.

**Parameter**	**Value / Description**
Time step Δt	0.1 days
Simulation duration	1950–2025
Initial condition x_0	0.2
Carrying capacity K	1.0
Growth rate range $(r_{\text{start}}, r_{\text{end}})$	$(-0.5, 0.5)$
Transition midpoint	0.65 of total time
Transition width	1/8 of total duration
Noise amplitude σ	0.03
Seasonal amplitude A_{seasonal}	0.05
Seasonal frequency ω	365 days
Integration scheme	Euler–Maruyama

Table S3: Parameters used in the logistic time series model.

Spatio-Temporal Vegetation Models

For the reaction-diffusion spatial vegetation model, we simulate 20 years of data with a $dt = 0.1$ days, saving the vegetation state at the end of each day. This data is used in Figure 3 and Supplemental Figure S6.

**Parameter**	**Value / Description**
Grid size	50×50
Time step Δt	0.1 days
Simulation duration	20 years
Base growth rate r_v	0.5
Vegetation half-saturation constant h_v	0.2
Environmental half-saturation constant h_E	2.0
Nonlinearity exponent p	4.0
Diffusion coefficient D	0.5
Noise amplitude σ	0.02
Initial environmental forcing $E_{0,\text{start}}$	4.0
Final environmental forcing $E_{0,\text{end}}$	8.0
Seasonal amplitude in E_0 (A_{E_0})	0.15
Mean seasonal amplitude in r_v ($A_{r_v,\text{mean}}$)	0.15
Std. of amplitude heterogeneity ($A_{r_v,\text{std}}$)	0.03
Max spatial phase shift	$\pi/6$ ($\sim$ 30 days)
Seasonal period T_{season}	365 days
Integration scheme	Euler–Maruyama

Table S4: Parameters used in the spatial reaction-diffusion vegetation model.

For the Klausmeier spatial pattern formation model, we simulate 20 years of data with a $dt = 0.01$ days, saving the snapshots of vegetation (N) and water (W) state at the end of each day. This data is used in Supplemental Figure S7.

**Parameter**	**Value / Description**
Grid size	50×50
Time step Δt	0.01 days
Simulation duration	20 years
Vegetation mortality m	0.45
Vegetation diffusion D_n	1.0
Water diffusion e	20.0
Noise amplitude σ	0.01
Initial rainfall a_{start}	1.2
Final rainfall a_{end}	0.65
Seasonal amplitude A_{seasonal}	0.02
Seasonal period T_{season}	365 days
Integration scheme	Euler–Maruyama

Table S5: Parameters used in the seasonally-forced pattern formation model.
